# Captivity results in disparate loss of gut microbial diversity in closely related hosts

**DOI:** 10.1093/conphys/cou009

**Published:** 2014-03-21

**Authors:** Kevin D. Kohl, Michele M. Skopec, M. Denise Dearing

**Affiliations:** 1 Department of Biology, University of Utah, Salt Lake City, UT 84112, USA; 2 Department of Zoology, Weber State University, Ogden, UT 84408, USA

**Keywords:** Captive breeding, captivity, dietary specialization, host–microbe interactions, microbial diversity, *Neotoma*

## Abstract

The gastrointestinal tracts of animals contain diverse communities of microbes that provide a number of services to their hosts. There is recent concern that these communities may be lost as animals enter captive breeding programmes, due to changes in diet and/or exposure to environmental sources. However, empirical evidence documenting the effects of captivity and captive birth on gut communities is lacking. We conducted three studies to advance our knowledge in this area. First, we compared changes in microbial diversity of the gut communities of two species of woodrats (*Neotoma albigula*, a dietary generalist, and *Neotoma stephensi*, which specializes on juniper) before and after 6–9 months in captivity. Second, we investigated whether reintroduction of the natural diet of *N. stephensi* could restore microbial diversity. Third, we compared the microbial communities between offspring born in captivity and their mothers. We found that the dietary specialist, *N. stephensi*, lost a greater proportion of its native gut microbiota and overall diversity in response to captivity compared with *N. albigula*. Addition of the natural diet increased the proportion of the original microbiota but did not restore overall diversity in *N. stephensi*. Offspring of *N. albigula* more closely resembled their mothers compared with offspring–mother pairs of *N. stephensi*. This research suggests that the microbiota of dietary specialists may be more susceptible to captivity. Furthermore, this work highlights the need for further studies investigating the mechanisms underlying how loss of microbial diversity may vary between hosts and what an acceptable level of diversity loss may be to a host. This knowledge will aid conservation biologists in designing captive breeding programmes effective at maintaining microbial diversity.

Sequence Accession Numbers: NCBI's Sequence Read Archive (SRA) – SRP033616

## Abstract (precis)

We compared microbial community structure in two rodent species before and after captivity. One species lost a greater proportion of diversity in captivity, which was not rescued when they were returned to their natural diet. Mothers effectively transmitted microbiota to their offspring. These results have implications for captive breeding programs.

## Introduction

The gut microbial communities of animals are hyperdiverse and influence many aspects of their physiology, such as nutrition, immune development and even behaviour (Amato, 2013). The preservation of the microbial diversity present in the gut is thought to be critical to the success of their hosts (Redford *et al.*, 2012). For instance, loss of microbial diversity may underlie increased disease prevalence in humans by resulting in microbial communities that are more susceptible to invasion or by altering host immune function (Blaser and Falkow, 2009). Additionally, gut microbes serve as sources of novel gene products, such as enzymes for biomass degradation (Hess *et al.*, 2011) or bioremediation (Verma *et al.*, 2006).

There is concern that bringing animals into captivity for breeding programmes may result in a loss of microbial diversity, which may contribute to the failures of reintroduced individuals (Redford *et al.*, 2012). For instance, loss of the nutritional, immunological or behavioural functions provided by microbes (Amato, 2013) may result in a reduction in host fitness. However, there have been few empirical studies tracking the loss of microbial diversity when animals are brought into captivity. Moreover, the transmission of microbes from mother to offspring for a wild species held in captivity has not been well characterized. The birthing process represents the greatest microbial acquisition event in the lives of many species (Palmer *et al.*, 2007; Dominguez-Bello *et al.*, 2010; Funkhouser and Bordenstein, 2013). It is currently unknown whether the uniform conditions of captivity tend to homogenize microbial gut communities among individuals or species.

We addressed this gap in knowledge using woodrats (*Neotoma* spp.), which are small, herbivorous rodents that serve as a model system to study interactions between dietary plant toxins and the gut microbiota (Dearing *et al.*, 2000). Woodrats are interesting from a microbial perspective because they maintain dense microbial populations in the foregut of their stomach, as well as their caecum (Kohl *et al.*, 2014). We have previously demonstrated that the desert woodrat (*Neotoma lepida*) retained a majority of its gut microbes after 6 months in captivity and shared more microbial species with wild conspecifics than other studies on captive-bred species (Kohl and Dearing, 2014). However, in that study, *N. lepida* exhibited a substantial loss in microbial diversity. It is unclear how consistent captivity-induced changes in microbial diversity are across host species. Furthermore, diet greatly influences microbial community diversity (Ley *et al.*, 2008; Turnbaugh *et al.*, 2009b; Kohl and Dearing, 2012), and thus returning animals to their natural diet in captivity may rescue microbial diversity.

Here, we compared the gut microbial diversity of two woodrat species, the white-throated woodrat (*Neotoma albigula*) and Stephens' woodrat (*Neotoma stephensi*), in the wild and in captivity. Both species were held in identical environmental conditions and fed the same diet, allowing us to determine whether species maintain unique microbial signatures in captivity. *Neotoma albigula* is a generalist species that feeds on a variety of plants across its range, such as *Opuntia* cactus, yucca, juniper, other shrubs and grasses (Macedo and Mares, 1988). Conversely, *N. stephensi* is a dietary specialist that consumes a diet of 60–95% juniper (*Juniperus monosperma*; Vaughn, 1982; Dial, 1988). Given that diet influences microbial community composition over an evolutionary time scale (Muegge *et al.*, 2011), we predicted that the specialist species would lose more microbial diversity due to the novel diet in captivity.

The level of specialization exhibited by *N. stephensi* also permitted the recreation of its natural diet in captivity. In *N. lepida*, roughly a quarter of the bacterial species found in the gut are also found on plant surfaces, and so plants may represent a small microbial source to the gut community (Kohl and Dearing, 2014). Additionally, diet is known to impact the microbial community structure of the gut (Ley *et al.*, 2008; Turnbaugh *et al.*, 2009b; Kohl and Dearing, 2012). Therefore, we predicted that feeding on juniper in captivity would restore microbial diversity to resemble more closely the gut microbiomes of woodrats in nature.

We also investigated the transmission of gut microbes from mothers to offspring. Given that a large proportion of microbes are acquired from mothers through the birth process and milk feeding in mammals (Palmer *et al.*, 2007; Dominguez-Bello *et al.*, 2010; Funkhouser and Bordenstein, 2013), we predicted that offspring would resemble their own mothers and species, thus suggesting that captive breeding programmes may retain wild microbiomes.

Finally, we compared food intakes between wild-caught and captive-born animals as an indicator of microbial function. Differences in food intake could be indicative of changes in fibre digestibility (Veloso and Bozinovic, 1993) or tolerance to the toxins present in juniper (Dearing *et al.*, 2000); two functions provided by the gut microbiota (Freeland and Janzen, 1974; Stevens and Hume, 2004). We predicted that captive-born animals would perform as well as wild-caught individuals due to effective microbial transmission.

## Methods

### Effect of captivity on the microbiota of two woodrat hosts

White-throated woodrats (*N. albigula*, *n* = 6) were collected from Castle Valley, UT, USA, in two groups (December 2011 and March 2012). For all analyses, we initially compared these two groups but found no differences, and thus they were combined. Stephens' woodrats (*N. stephensi*, *n* = 7) were collected near Wupatki National Monument, AZ, USA, in March 2012. Details of these trapping locations and durations in captivity are shown in Table 1. All animals were captured using Sherman live traps baited with apple, peanut butter and oats. Faeces were collected from traps the next morning and represent the ‘wild’ samples. Food is retained in the woodrat gut for >7.5 h (Kohl *et al.*, 2014), which was roughly the maximal length of time animals could have been in traps. Thus, the small amount of bait provided in traps was unlikely to impact the faecal microbial communities of woodrats during the trapping procedure. Animals were immediately transported to Weber State University, housed individually in shoebox cages with wood shavings, and fed high-fibre rabbit chow (Harlan Teklad product #2031, Madison, WI, USA) *ad libitum* for 6–9 months (see Table 1). In September 2012, we measured body mass and food intake on a rabbit chow diet. Additionally, we collected faecal samples by placing animals in sterile cages for 2 h. Faeces were collected and frozen for later analysis, and will be referred to as ‘captive’ samples. The Weber State Institutional Animal Care and Use Committee approved all experimental techniques under protocol 11-02.

**Table 1:** Details of trapping locations and time in captivity

Animals	Trapping location	Date trapped	Time in captivity before sample collection
*Neotoma albigula*			
Group 1 (*n* = 3)	Castle Valley, UT, USA (38°30′N, 109°18′W)	December 2011	9 months
Group 2 (*n* = 3)	Castle Valley, UT, USA (38°30′N, 109°18′W)	March 2012	6 months
*Neotoma stephensi* (*n* = 7)	Wupatki National Monument, AZ, USA (35°30′ N, 111°27′ W)	March 2012	6 months

### Return to a natural diet

After 6 months in captivity, individuals of *N. stephensi* underwent a diet trial in which they were fed ground juniper (*J. monosperma*) to mimic their natural diet (4 days on 25%, 1 day on 50% and 4 days on 75%). Gut microbial communities respond rapidly to dietary changes (David *et al.*, 2013), and this length of time is sufficient to induce changes in woodrat microbial communities (Kohl and Dearing, 2012). Diets were prepared using methods described elsewhere (Skopec *et al.*, 2007). Food intakes and body masses were recorded daily. Faeces were collected on the last day of this feeding trial and represent ‘juniper diet’ samples.

### Transmission of microbes to offspring

Several animals (*N. albigula*, *n* = 2 and *N. stephensi*, *n* = 4) were pregnant when trapped and gave birth within 30 days of being brought into captivity. Offspring (*N. albigula*, *n* = 3 and *N. stephensi*, *n* = 5) were housed with their mothers until they were weaned at 2 months of age, placed in individual cages and fed rabbit chow. This time frame was similar to nature, where animals wean at 20–35 days old and first leave the den around 42 days old (Vaughn and Czaplewski, 1985; Macedo and Mares, 1988). Faeces were collected to inventory microbial communities of all offspring at least 4 months after weaning. Data from siblings were averaged to form one independent unit. Captive-born *N. stephensi* were subsequently fed a diet of 75% juniper to investigate whether they had the ability to consume this toxic, high-fibre diet (see details of feeding trial in the previous subsection).

### Bacterial inventories

Whole DNA was isolated from faeces using a QIAamp DNA Stool Mini Kit (Qiagen, Germantown, MD, USA). Extracted DNA was sent to Argonne National Laboratory (Lemont, IL, USA) for sequencing. Bacterial inventories were conducted by amplifying the V4 region of the 16S rRNA gene using primers 515F and 806R, and paired-end sequencing on an Illumina MiSeq platform (Caporaso *et al.*, 2012).

Sequences were analysed using the QIIME software package (Caporaso *et al.*, 2010). Sequences underwent standard quality control and were split into libraries using default parameters in QIIME. Sequences were grouped into *de novo* operational taxonomic units (OTUs) using UCLUST (Edgar, 2010) with a minimal sequence identity of 97%. The most abundant sequences within each OTU were designated as a ‘representative sequence’ and then aligned against Greengenes 13_5 (DeSantis *et al.*, 2006) using PyNAST (Caporaso *et al.*, 2009) with default parameters set by QIIME. A PH Lane mask supplied by QIIME was used to remove hypervariable regions from aligned sequences. FastTree (Price *et al.*, 2009) was used to create a phylogenetic tree of representative sequences. The OTUs were classified using the Ribosomal Database Project classifier with a the standard minimal support threshold of 80% (Wang *et al.*, 2007). Sequences identified as chloroplasts or mitochondria were removed from analysis.

The number of OTUs that were shared between samples (wild and captive or wild and juniper diet) was determined within species to calculate the relative loss and retention of OTUs. For this analysis, samples were pooled within a condition (wild, captive, juniper diet) in order to maximize detection of microbial OTUs within a given condition. The proportion of OTUs retained was calculated as total OTUs shared between conditions divided by the total number of OTUs in both conditions. Differences in the relative loss and retention of OTUs between species as well as between diets in *N. stephensi* were compared using χ^2^ analysis (Whitlock and Schluter, 2009).

Several α diversity measurements were calculated for each sample using QIIME. We calculated the Shannon diversity index (Shannon and Weaver, 1949), a biodiversity measure that incorporates both richness and evenness, and Faith's phylogenetic diversity (Faith, 1992), which measures the cumulative branch lengths from randomly sampling OTUs from each sample. For each sample, we calculated the mean of 20 iterations for a subsampling of 12 400 sequences. Diversity measurements were compared between species using repeated-measures ANOVA with sample (wild or captive) as the repeated measure and species as the factor using JMP 10.0. Student's paired *t*-tests were used to compare between conditions (captive vs. juniper diet) in *N. stephensi*.

We also compared community membership (presence or absence of bacterial lineages). We calculated unweighted UniFrac distances, which are the proportion of microbial OTUs that are specific to one sample or the other (Lozupone and Knight, 2005). UniFrac distances were compared using a Student's unpaired *t*-test (between species) or a repeated-measures ANOVA. We also conducted principal coordinates analysis (PCoA) on UniFrac scores to visualize similarities in microbial communities (Hamady *et al.*, 2010). Clustering by treatment or individuals was determined using the analysis of similarity test (ANOSIM) within QIIME. Relative abundances of taxa were compared similar to α diversity measurements. All *P*-values were Bonferroni corrected, with α = 0.05 as a threshold for significance. All sequences were deposited in NCBI's Sequence Read Archive (SRA) under accession SRP033616.

### Statistical analysis of food intake and body masses

We compared body mass and food intake as indicators of tolerance to experimental diets. Data were compared between species with two-way ANOVAs, with either food intake or body mass as the dependent variable and species and status (wild caught or captive born) as the independent factors using JMP 10.0. Food intakes and body masses of *N. stephensi* fed rabbit chow or 75% juniper were compared with repeated-measures ANOVAs, with either food intake or body mass on the two different diets as the repeated measure and status (wild caught or born in captivity) as the factor.

## Results

### Effect of captivity on the microbiota of two woodrat hosts

**Figure 1:**
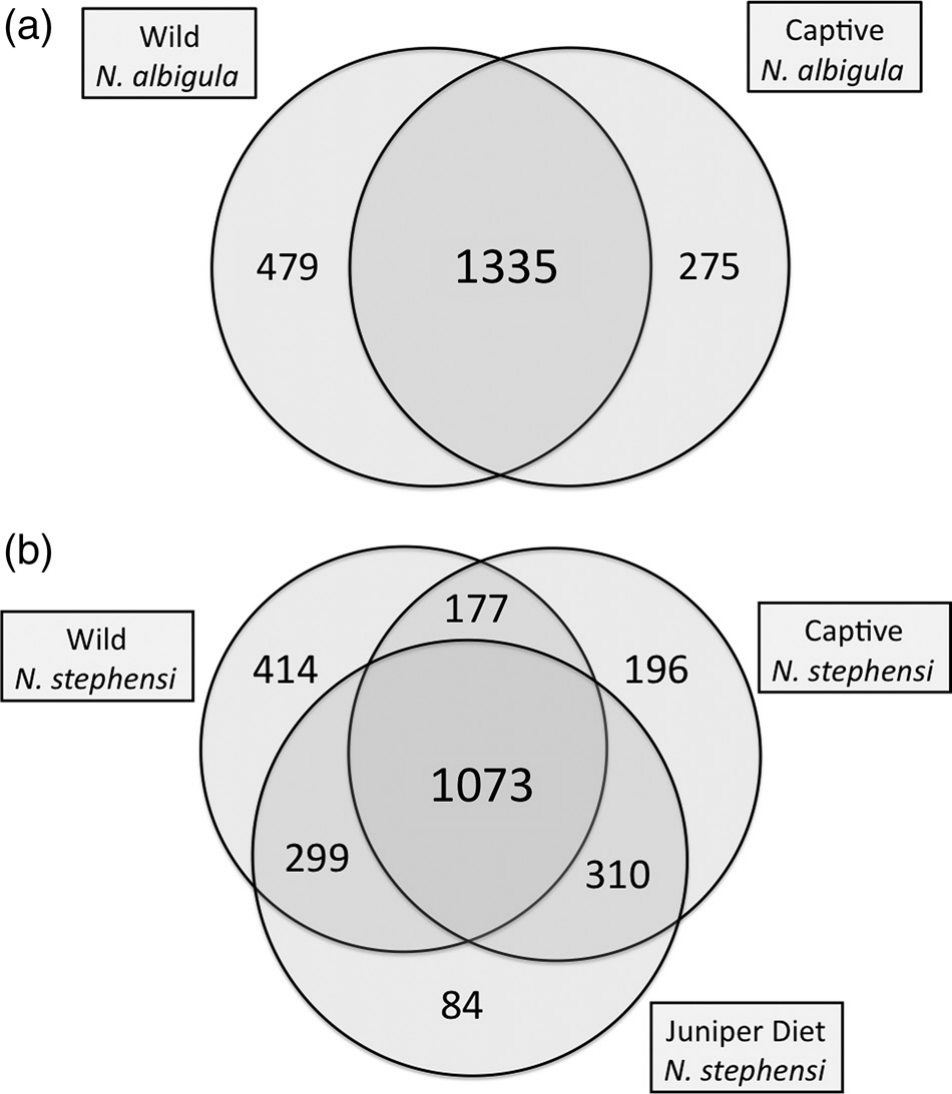
Shared microbial operational taxonomic units (OTUs) in the faeces of woodrats. (a) *Neotoma albigula* in the wild and after 6–9 months of captivity. (b) *Neotoma stephensi* in the wild, after 6 months in captivity fed rabbit chow diet, and fed a 75% juniper diet.

**Figure 2:**
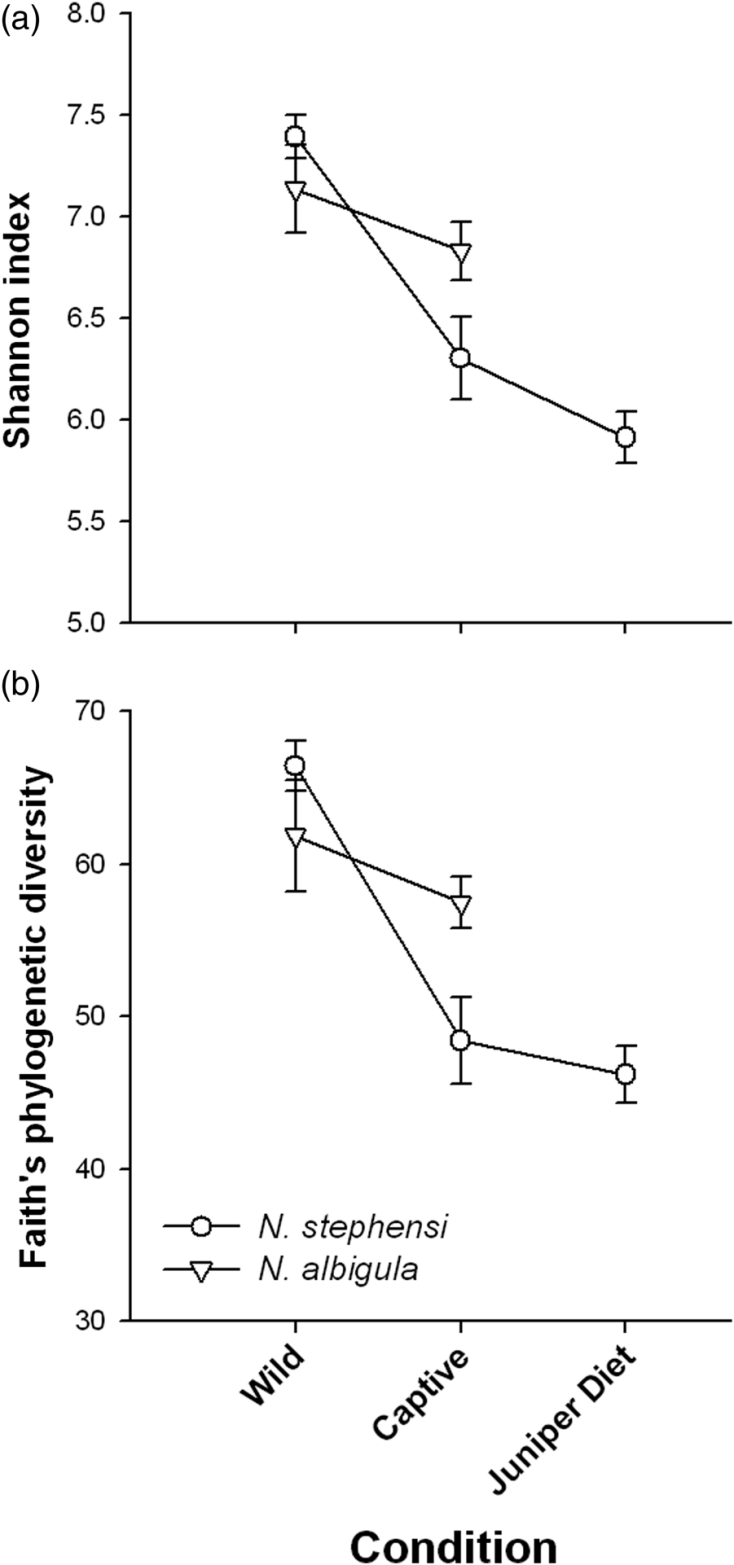
Diversity metrics of microbial communities from woodrats from the wild, after 6–9 months in captivity fed rabbit chow diet, and fed a 75% juniper diet. (**a**) Shannon diversity index. (**b**) Faith's phylogenetic diversity.

The microbiota of *N. albigula* and *N. stephensi* responded differently to captivity. First, the hosts differed in the relative loss and retention of OTUs after ≥6 months in captivity on a rabbit chow diet (χ^2^ = 81.2, *P* < 0.001). In *N. albigula*, 64% of microbial OTUs (1335 of 2089) were shared between samples collected from the wild and in captivity (Fig. 1a). Samples from *N. stephensi* collected from the wild and in captivity shared only 51% of OTUs (1250 of 2469; Fig. 1b). Diversity decreased significantly during captivity for both species (repeated-measures ANOVA; Shannon index, *F*_1,11_ = 13.43, *P* = 0.004; and Faith's phylogenetic diversity, *F*_1,11_ = 20.86, *P* = 0.0008; Fig. 2). Although there was no main effect of species (*P* > 0.3 for both metrics), there were significant or near-significant species-by-captivity interactions (species × captivity interaction: Shannon index, *F*_1,11_ = 4.25, *P* = 0.06; and Faith's phylogenetic diversity, *F*_1,11_ = 7.71, *P* = 0.018; Fig. 2). *Neotoma stephensi* exhibited a greater loss of overall diversity than *N. albigula* (Fig. 2).

**Figure 3:**
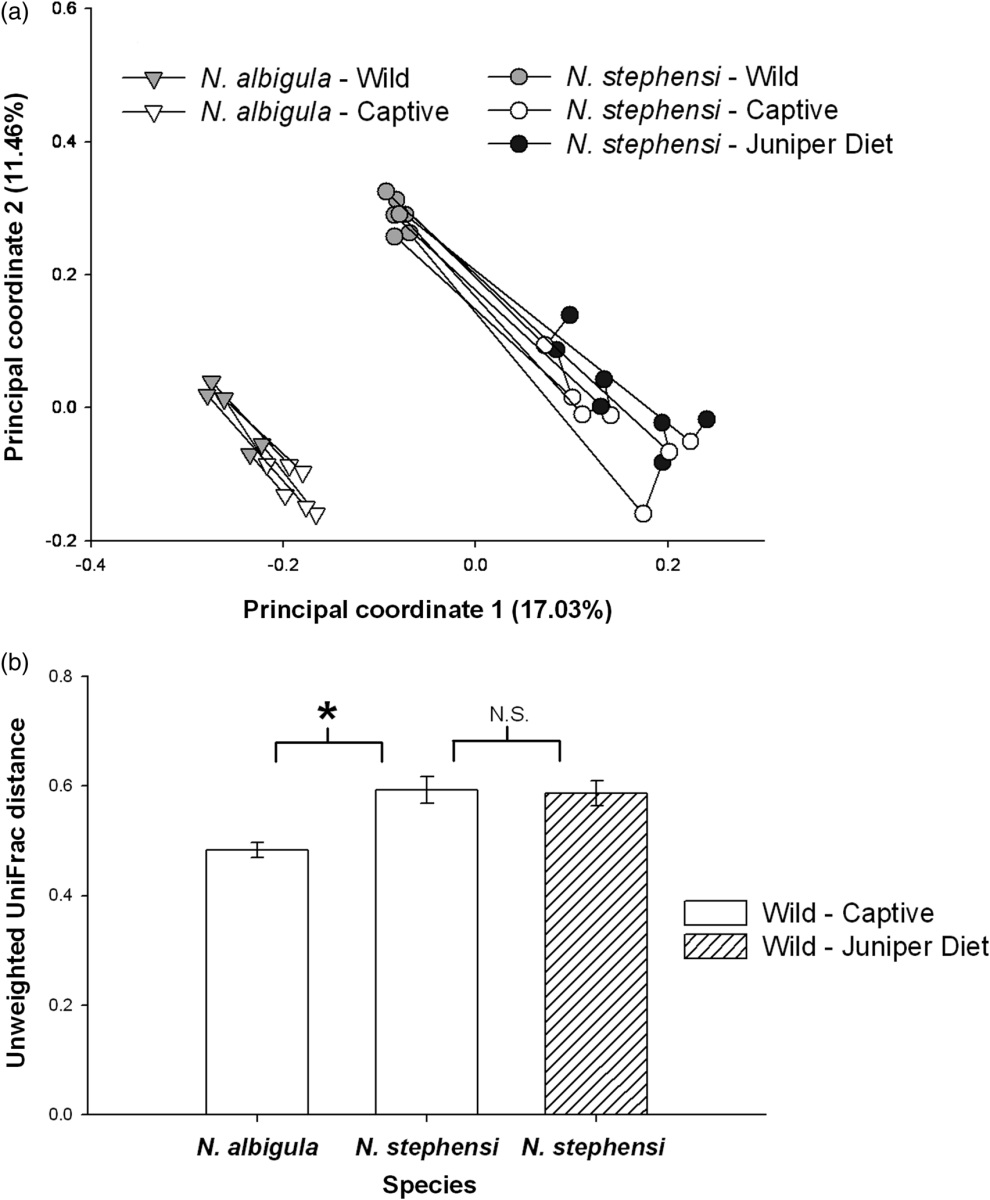
Changes in microbial communities between the wild and captivity. (**a**) Principal coordinates analysis. Lines connect an individual over time. (**b**) Unweighted UniFrac distances. Open bars represent mean distances between samples from the wild compared with animals in captivity. The hatched bar represents the distance between samples collected from animals in the wild to samples collected from animals fed the juniper diet. The asterisk denotes statistical significance between *N. albigula* and *N. stephensi*. The difference between *N. stephensi* fed commercial chow and 75% juniper was not significant (n.s.).

The woodrat species maintained unique microbial communities as determined by principal co-ordinates analysis (Fig. 3a). Faecal communities clustered according to species and captivity status (ANOSIM, *P* < 0.001 for both; Fig. 3a). These results are also shown by UniFrac distances, which are the fraction of branch lengths on a phylogenetic tree of all microbial OTUs that are unshared by two samples. UniFrac distances between faecal samples of *N. albigula* from the wild and in captivity were significantly smaller than in *N. stephensi* (Student's unpaired *t*-test, *P* = 0.003; Fig. 3b). This result shows that the microbial communities of *N. albigula* from the wild and in captivity were more similar than in *N. stephensi*.

**Figure 4:**
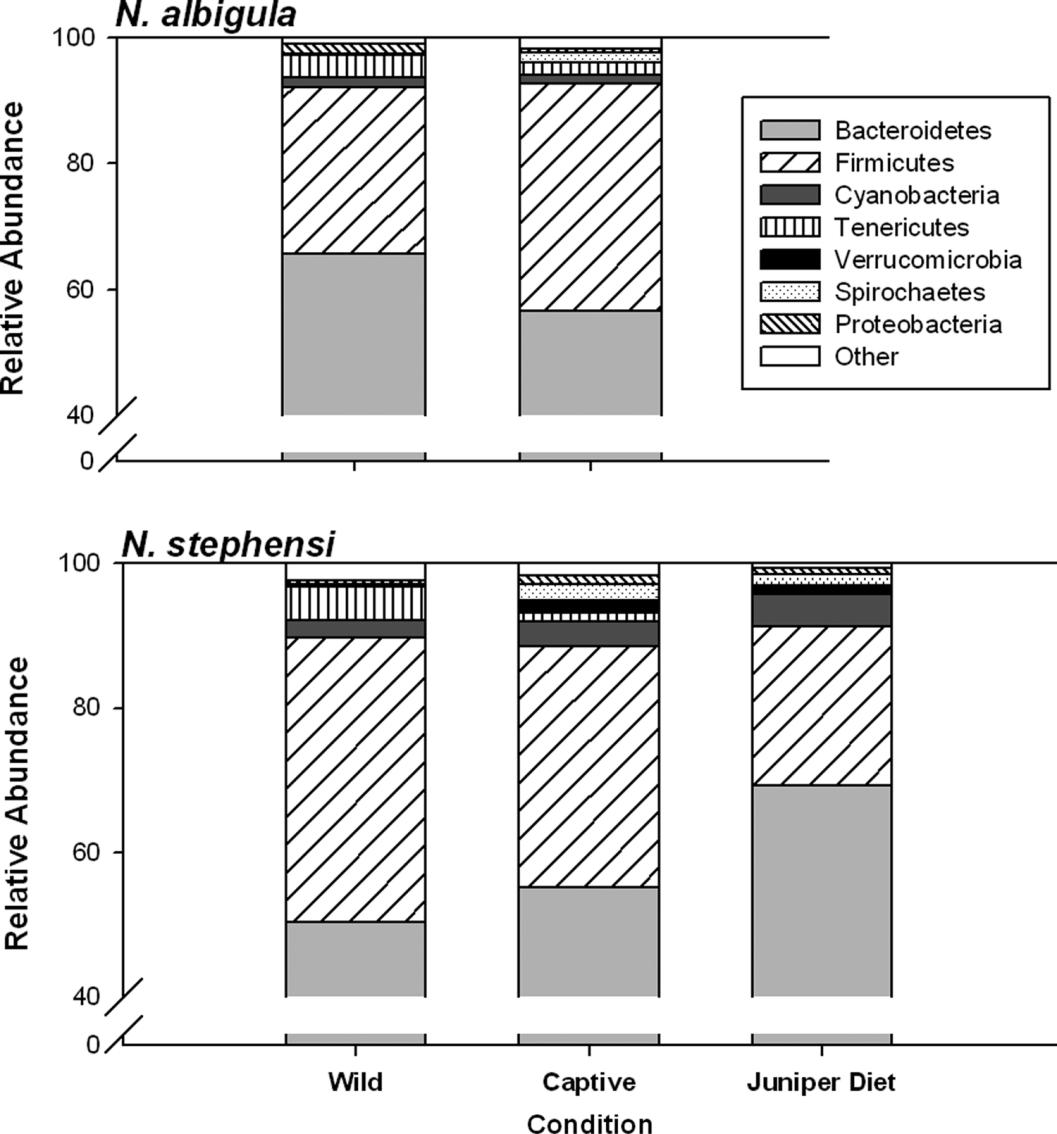
Relative abundances of microbial phyla in faecal samples of woodrats collected from the wild, after 6–9 months in captivity fed rabbit chow diet, and fed a 75% juniper diet.

These results were driven by differential abundances of a number of microbial phyla. *Neotoma stephensi* harboured a greater relative abundance of Cyanobacteria compared with *N. albigula* (Fig. 4 and Table 2). Furthermore, the phylum Verrucomicrobia was detected in all *N. stephensi* individuals, but was absent from all *N. albigula* (Fig. 4 and Table 2). Captivity resulted in a decrease in the relative abundance of Tenericutes in both host species and an increase in the abundance of Verrucomicrobia in *N. stephensi* (Fig. 4 and Table 2). The differential effect of captivity on microbial community structure is driven by contrasting responses in microbial phyla. For example, the abundance of Firmicutes increased in captivity in *N. albigula* but decreased in *N. stephensi*, while the abundance of Proteobacteria exhibited the opposite response (Fig. 4 and Table 2).

**Table 2:** Repeated-measures ANOVA results for relative abundances of bacterial phyla in faecal samples of two species of woodrats collected in the wild and captivity

Bacterial phyla	*F*	d.f.	*P*-value
Firmicutes			
Species	1.81	1,11	0.21
Captivity	0.23	1,11	0.64
Species × captivity	5.34	1,11	0.04*
Cyanobacteria			
Species	4.91	1,11	0.04*
Captivity	0.77	1,11	0.39
Species × captivity	0.86	1,11	0.37
Tenericutes			
Species	0.07	1,11	0.79
Captivity	11.23	1,11	0.006*
Species × captivity	1.45	1,11	0.25
Verrucomicrobia			
Species	17.96	1,11	0.001*
Captivity	10.33	1,11	0.008*
Species × captivity	10.33	1,11	0.008*
Proteobacteria			
Species	0.53	1,11	0.48
Captivity	0.97	1,11	0.35
Species × captivity	8.70	1,11	0.01*

*P < 0.05.

### Return to a natural diet

The addition of juniper to the diet of *N. stephensi* significantly increased the proportion of OTUs shared with samples collected in the wild to 58.2% (1372 of 2357 OTUs) from 51% before the diet amendment (Fig. 1b; χ^2^ = 27.9, *P* < 0.001). *Neotoma stephensi* exhibited a slight decrease in the Shannon index after being fed the juniper diet (Student's paired *t*-test between 0 and 75% juniper samples, *P* = 0.06), but showed no difference in Faith's phylogenetic diversity (*P* = 0.15; Fig. 2). Samples collected from individuals fed the juniper did not cluster differentially from ‘captivity’ samples collected when animals were fed rabbit chow (Fig. 3a). *Neotoma stephensi* did not exhibit a significant decrease in UniFrac distance when fed juniper (Fig. 3b).

Feeding on the juniper diet did not cause relative abundances of microbial phyla to match those of samples collected from the wild. The relative abundances of five microbial phyla were significantly different between samples collected from the wild and collected from the juniper diet treatment (Fig. 4 and Table 3). Rather, samples collected during the juniper diet treatment exhibited greater similarity to samples collected during captivity, except for an increase in the abundance of Firmicutes (Fig. 4 and Table 3).

**Table 3:** Statistics of pair-wise comparisons of relative abundances of various bacterial phyla in the faeces of *N. stephensi*

Bacterial phyla	Wild vs. juniper diet	Captive vs. juniper diet
Bacteroidetes	0.003*	0.04*
Firmicutes	0.001*	n.s.
Cyanobacteria	n.s.	n.s.
Tenericutes	0.001*	n.s.
Verrucomicrobia	0.016*	n.s.
Spirochaetes	0.04*	n.s.
Proteobacteria	n.s.	n.s.

*P*-values have been Bonferroni corrected.

*P < 0.05; n.s., not significant.

### Transmission to offspring

**Figure 5:**
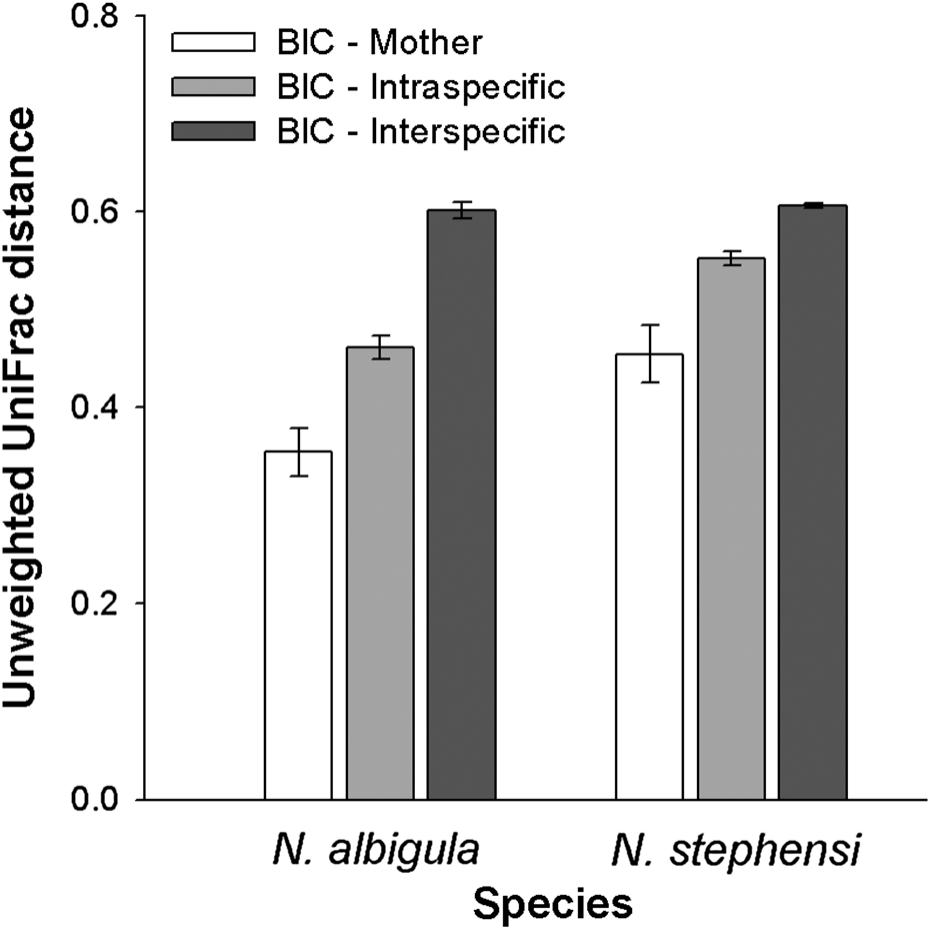
UniFrac distances between individuals born in captivity (BIC) and others. Open bars compare offspring with their mothers. Light grey bars compare offspring with adult members of the same species (excluding their mother). Dark grey bars compare offspring with adult members of the other species.

Analysis of faecal samples of captive-born individuals compared with their mothers confirmed that the microbiota is transmitted from mothers to offspring. UniFrac distances between mothers and offspring were smaller than between offspring and other individuals of the same species or different species (repeated-measures ANOVA; within-subjects effect, *F*_1,4_ = 17.22, *P* = 0.014; Fig. 5). Overall, *N. albigula* exhibited enhanced microbial transmission from mother to offspring, as their UniFrac distances were smaller than *N. stephensi* (species effect, *F*_1,4_ = 16.57, *P* = 0.015; Fig. 5). Additionally, offspring more closely resembled their species than interspecifics (within-subjects effect, *F*_1,4_ = 122.64, *P* = 0.0004), with *N. albigula* maintaining their species signature better than *N. stephensi* (species effect, *F*_1,4_ = 73.94, *P* = 0.001). There were no differences in the relative abundances of microbial taxa between adults in captivity and offspring. Interestingly, all offspring of *N. albigula* lacked the phylum Verrucomicrobia, similar to the difference seen in wild-caught adults, further demonstrating that species-specific microbial communities were transmitted to offspring.

### Food intake and body mass as an indicator of function

Wild-caught and captive-born animals had similar body masses and food intakes when fed rabbit chow (Table 4). Captive-born *N. stephensi* consumed similar amounts of rabbit chow and the 75% juniper diet compared to the wild-caught animals (*F*_1,10_ = 1.82, *P* = 0.2). All *N. stephensi* consumed more of the 75% juniper diet than rabbit chow (diet effect, *F*_1,10_ = 6.98, *P* = 0.025).

**Table 4:** Mean ± SEM body masses and food intake of wild-caught and captive-born woodrats

Animals	Rabbit chow		75% juniper	
	Body mass (g)	Food intake (g/g body mass)	Body mass (g)	Food intake (g/g body mass)
*Neotoma albigula*				
Wild caught (*n* = 6)	180.29 ± 9.88	0.06 ± 0.004		
Captive born (*n* = 3)	179.67 ± 12.81	0.05 ± 0.011		
*Neotoma stephensi*				
Wild caught (*n* = 7)	169.29 ± 7.71	0.06 ± 0.006	163.29 ± 7.62	0.08 ± 0.003
Captive born (*n* = 5)	196.40 ± 9.50	0.07 ± 0.002	189.8 ± 12.00	0.08 ± 0.004

*Neotoma albigula* were not fed a diet of 75% juniper.

## Discussion

Conservation of gut microbial communities has been largely overlooked by conservation biologists, despite their importance in animal health and performance (Redford *et al.*, 2012). Here we show that captivity can have disparate effects on the microbiomes of closely related hosts. We discuss a number of potential mechanisms driving these results, which will require further research to understand the effects of captivity on the loss of microbial diversity.

The loss of diversity in captivity may be driven partly by the loss of environmental sources of microbes. Both woodrat species exhibited a decrease in microbial diversity and a decrease in the relative abundance of the phylum Tenericutes. These trends have also been shown in *N. lepida* (Kohl and Dearing, 2014). Animals housed in captivity experience a much cleaner environment than in the wild. In nature, woodrats live in stick structures called middens that are passed on from generation to generation (Betancourt *et al.*, 1990). While we did not inventory the microbial environment of middens, they are likely to be a rich source of microbes, given that unrelated individuals of woodrats and other species, such as mice, snakes and ground squirrels, may inhabit middens (Betancourt *et al.*, 1990; Baxter *et al.*, 2009). Additionally, woodrats frequently collect and store scat from jackrabbits, coyotes and cows in or on their middens (Betancourt *et al.*, 1990). Repeated exposure to faecal material from other individuals and species may increase the microbial diversity of woodrats in nature. Once in captivity, inter- and intraspecies inoculation no longer occurs (except for interactions with researchers), and woodrats receive very few microbes from environmental sources in captivity, such as food and bedding (Kohl and Dearing, 2014). The removal from dense microbial sources may partly explain the observed loss of diversity, though does not explain species-specific differences.

Captivity had a markedly different effect on the microbiomes of *N. albigula* and *N. stephensi*. Individuals of *N. albigula* retained a majority (64%) of their microbial OTUs after being held in captivity for 6–9 months. These results are similar to findings in captive *N. lepida*, which also shared 64% of their microbial OTUs with samples collected from free-ranging individuals (Kohl and Dearing, 2014). However, *N. stephensi* shared only 50% of OTUs between samples taken before and after captivity. Likewise, both *N. lepida* and *N. albigula* exhibited a ∼7% decrease in Faith's phylogenetic diversity in captivity, while *N. stephensi* exhibited a 27% loss. These results are especially noteworthy given that some individuals of *N. albigula* had been in captivity for 3 months longer than *N. stephensi* (see Table 1). Many closely related species exhibit different responses to captivity in terms of stress physiology and immune function (Mason, 2010). These physiological responses to captivity may drive the differential retention or loss of microbes.

Another factor that may underlie the differences between species is the high degree of dietary specialization exhibited by *N. stephensi*. There is strong coevolution between diet and gut microbial communities that seems to be driven by the relative amounts of various nutrients that animals consume (Ley *et al.*, 2008; Muegge *et al.*, 2011). Therefore, the microbes of dietary specialists may be especially reliant on the types of nutrients consumed by specialist hosts and more susceptible to perturbation by a novel diet. Specialist herbivores are often difficult to maintain in captivity (Shipley *et al.*, 2009), which may be due to loss of microbial diversity. Conversely, dietary specialists may not need to maintain as much microbial diversity as generalists in order to maintain function, due to the limited diversity in their diet. Indeed, one dietary specialist, the giant panda (*Ailuropoda melanoleuca*) exhibits the lowest amount of microbial diversity of any mammal studied to date (Zhu *et al.*, 2011). The *N. stephensi* in our study were still able to specialize on juniper despite reductions in diversity due to captivity. Future research on the microbial communities of dietary specialists in the wild and in captivity will uncover whether this is a general trend.

Our results suggest that diet is not the sole driver of loss of microbial diversity in captivity. Both species in this study were placed on an identical diet of rabbit chow in captivity. If diet played a key role in sculpting microbial communities then it would be expected that the microbial communities of various woodrat species would converge with one another after being exposed to a similar diet and thus, unique species signatures would be lost. This outcome did not occur; the microbial signatures of woodrats in captivity continued to cluster together by species when measured by principal coordinate analysis. Moreover, returning *N. stephensi* to its natural diet did not fully restore microbial diversity to pre-captivity levels or cause shifts in the relative abundances of microbial taxa to match those of wild samples. However, the addition of juniper did result in the return of 299 OTUs found in the samples collected in the wild. These microbial OTUs may have come from juniper foliage or they may have been present in undetectable levels when animals were fed rabbit chow. Future studies could investigate whether microbial diversity is better retained when animals are fed a natural diet immediately when brought into captivity.

The microbiota of offspring born in captivity closely resembled those of their mothers. Vertical transmission of microbes has been documented in a number of mammalian species, which acquire their microbes through contact with faecal and vaginal microbes during the birth process and also through milk (Palmer *et al.*, 2007; Dominguez-Bello *et al.*, 2010; Funkhouser and Bordenstein, 2013). We compared vertical transmission across species born in the same captive environment. We found that offspring of *N. albigula* more closely resemble their mothers compared with *N. stephensi*; this result suggests enhanced vertical transmission for *N. albigula*. The mechanisms and limitations of vertical transmission of the microbiota in mammals are poorly understood (Palmer *et al.*, 2007; Dominguez-Bello *et al.*, 2010; Funkhouser and Bordenstein, 2013), and thus future research is required to determine what drives species-specific transmission.

Despite losses of diversity in captivity and between generations, woodrats nonetheless maintained a high proportion of their natural microbiota. Although the offspring of *N. albigula* and *N. stephensi* in this study were born and housed in the same room, they retained the unique microbial signatures characteristic of their species. For example, all *N. albigula* (born in captivity and wild caught) lacked the phylum Verrucomicrobia. These results demonstrate that captivity does not homogenize microbial communities between species.

It is worth noting that this study only monitored losses in taxonomic diversity and did not investigate functional diversity of the microbiota. In humans, despite high variability in taxonomic diversity of microbes among individuals, there is high redundancy in functional gene content (Turnbaugh *et al.*, 2009a). Captive *N. stephensi* were able to ingest high levels of toxic, fibre-rich food and maintain mass in captivity, suggesting that the microbiota is still functional. Furthermore, captive-born *N. stephensi* still inherited a high tolerance for juniper and consumed similar amounts of the 75% juniper diet to the wild-caught *N. stephensi*. Thus, loss of microbial diversity in captivity may not necessarily result in a measureable loss of function.

Our results may have relevance for another species of woodrat, the Key Largo woodrat (*Neotoma floridana smalli*), which is federally endangered and has undergone a captive breeding programme (Alligood *et al.*, 2011). This programme was unsuccessful due to high predation rates on reintroduced individuals (McCleery *et al.*, 2013). Microbial diversity has been implicated in the movement patterns of laboratory rodents. For example, germ-free mice (those lacking a gut microbiota) voluntarily cover more area in an open arena, engage in more risky behaviours and exhibit lower indicators of anxiety (Heijtz *et al.*, 2011). It is possible that captive-bred Key Largo woodrats harbour lower microbial diversity, which may cause them to engage in more risky behaviours and experience higher rates of predation. However, this connection is largely speculative at this time.

Collaborations with zoos or wildlife refuges may be necessary to investigate how conditions in captivity impact the loss of gut microbial diversity and how this loss affects animal performance. Identification of the physiological or ecological mechanisms that drive differential loss of diversity between host species will inform conservation biologists about how best to design captive breeding programmes. Additionally, it would be ideal to compare microbial diversity of native and captive-bred individuals after being released into the wild. These studies will better inform scientists how to conserve taxonomic and functional diversity of the gut microbiome.
